# Choosing the Platinum Partner in Advanced Biliary Tract Cancer: A Propensity Score–Matched Real-World Comparison of Gemcitabine Plus Carboplatin Versus Gemcitabine Plus Cisplatin

**DOI:** 10.3390/life16071150

**Published:** 2026-07-11

**Authors:** Jirapat Wonglhow, Patrapim Sunpaweravong, Chirawadee Sathitruangsak, Arunee Dechaphunkul, Panu Wetwittayakhlang

**Affiliations:** 1 Division of Medical Oncology, Department of Internal Medicine, Faculty of Medicine, Prince of Songkla University, Songkhla 90110, Thailand; jirapat.jw@gmail.com (J.W.); spatrapi@medicine.psu.ac.th (P.S.); sjirawadee@gmail.com (C.S.); dr.arunee@gmail.com (A.D.); 2 Gastroenterology and Hepatology Unit, Division of Internal Medicine, Faculty of Medicine, Prince of Songkla University, Songkhla 90110, Thailand

**Keywords:** cholangiocarcinoma, gallbladder cancer, biliary tract cancer, chemotherapy, platinum, carboplatin, cisplatin, survival, efficacy

## Abstract

Gemcitabine plus cisplatin (GemCis) has been served as an important first-line chemotherapy backbone for unresectable locally advanced or metastatic biliary tract cancer (BTC). However, cisplatin is unsuitable for all patients. Gemcitabine plus carboplatin (GemCarbo) is frequently used as an alternative, but direct comparative data remain limited. We retrospectively review patients with unresectable locally advanced or metastatic BTC who received first-line GemCis or GemCarbo at Songklanagarind Hospital between 2011 and 2025. The primary endpoint was overall survival (OS). Secondary endpoints included progression-free survival (PFS), objective response rate (ORR), disease control rate (DCR), and selected laboratory-based safety outcomes. Propensity score matching was applied to reduce baseline treatment selection bias. Among 154 eligible patients, 95 received GemCis and 59 received GemCarbo. In the overall cohort, median OS was 8.44 months with GemCarbo and 9.82 months with GemCis (HR, 1.18; 95% CI, 0.82–1.70; *p* = 0.382). After propensity score matching, median OS was 8.44 months with GemCarbo and 11.63 months with GemCis (HR, 1.26; 95% CI, 0.84–1.90; *p* = 0.271). Median PFS was 4.27 and 5.75 months (HR, 0.91; 95% CI, 0.62–1.33; *p* = 0.617). ORR and DCR were comparable among evaluable patients. Increased serum creatinine was more frequent with GemCis, whereas hematologic toxicities were comparable. GemCarbo showed no statistically significant difference in OS or PFS compared with GemCis, suggesting that it may be considered as an alternative when cisplatin is unsuitable. However, prospective validation is warranted.

## 1. Introduction

Biliary tract cancers (BTCs) are aggressive malignancies associated with substantial global mortality [1,2]. The incidence of BTC varies markedly across geographic regions, with the highest rates observed in parts of Asia, including Thailand, where liver fluke–associated cholangiocarcinoma has historically contributed to a substantial disease burden [2,3,4]. Despite advances in diagnostic imaging, surgical techniques, and supportive care, a large proportion of patients are diagnosed with unresectable locally advanced or metastatic disease, for which systemic therapy remains the cornerstone of treatment [3,5,6].

For more than a decade, gemcitabine plus cisplatin (GemCis) has been established as the reference first-line regimen for advanced BTC, largely based on the ABC-02 phase III trial, which demonstrated improved survival with GemCis compared with gemcitabine alone [7]. More recently, the treatment landscape has evolved with the incorporation of immune checkpoint inhibitors into the GemCis backbone. In TOPAZ-1, durvalumab plus GemCis improved overall survival (OS) compared with GemCis alone [8,9,10], whereas KEYNOTE-966 similarly demonstrated an OS benefit with pembrolizumab plus GemCis [11]. These findings have led contemporary clinical practice guidelines to recommend chemoimmunotherapy with GemCis plus durvalumab or pembrolizumab as first-line treatment for eligible patients [5,6]. Nevertheless, access to immunotherapy remains variable across healthcare settings, and gemcitabine-platinum chemotherapy continues to be widely used as a key treatment backbone for patients with advanced BTC.

However, a major challenge in routine clinical practice is that not all patients are suitable candidates for cisplatin therapy. Baseline renal impairment, frailty, poor performance status, pre-existing neuropathy or hearing impairment, and the need for intensive hydration may limit the feasibility of cisplatin-based treatment [12,13]. In these circumstances, carboplatin is commonly substituted for cisplatin in combination with gemcitabine (GemCarbo) to maintain antitumor activity while improving treatment feasibility in patients who are less suitable for cisplatin. However, evidence supporting GemCarbo in advanced BTC remains limited and consists mainly of small studies and retrospective series often restricted to cisplatin-ineligible populations [14,15]. Direct comparative data between GemCarbo and GemCis in broader real-world cohorts remain sparse. Evidence from other malignancies suggests that carboplatin may provide comparable efficacy in some palliative settings [16,17], while cisplatin remains preferred in selected diseases or treatment contexts [18]. Therefore, disease-specific data are needed before extrapolating the relative efficacy of carboplatin and cisplatin to advanced BTC.

Clarifying whether GemCarbo provides outcomes comparable to those of GemCis is clinically relevant, particularly when treatment selection must balance efficacy, patient fitness, organ function, and accessibility. As patients receiving GemCarbo in routine practice may differ systematically from those receiving GemCis, comparative analyses should account for potential baseline imbalances between treatment groups. Therefore, this study aimed to compare the real-world clinical outcomes of first-line GemCarbo versus GemCis in patients with unresectable locally advanced or metastatic BTC, using propensity score–matched analysis.

## 2. Materials and Methods

### 2.1. Study Participants

This retrospective cohort study included patients with BTC treated at Songklanagarind Hospital, Prince of Songkla University, from January 2011 to December 2025. Eligible patients were aged 18 years or older with histologically confirmed adenocarcinoma arising from the biliary tract, including intrahepatic cholangiocarcinoma, extrahepatic cholangiocarcinoma, gallbladder cancer, or ampulla of Vater cancer. Patients were required to have unresectable locally advanced or metastatic disease and to have received first-line palliative chemotherapy with either GemCis or GemCarbo. Patients who had previously received systemic therapy for advanced disease were excluded. Prior adjuvant chemotherapy was permitted if it had been completed more than 6 months before the start of first-line palliative treatment.

Clinical information was obtained from electronic medical records through the Songklanagarind Hospital information system. Baseline variables collected at the initiation of first-line chemotherapy included age, sex, body mass index (BMI), Eastern Cooperative Oncology Group performance status (ECOG PS), history of cirrhosis, viral hepatitis status, primary tumor site, extent and sites of metastasis, and relevant laboratory parameters.

The study was approved by the Human Research Ethics Committee, Faculty of Medicine, Prince of Songkla University (REC.69023141). The requirement for informed consent was waived because of the retrospective design. All patient-level data was anonymized before analysis.

### 2.2. Treatment

Treatment selection and dose modification were determined by the treating physician according to institutional practice and individual patient factors. In the GemCis group, cisplatin was given at 25 mg/m^2^ intravenously, followed by gemcitabine 1000 mg/m^2^ on days 1 and 8 of a 21-day cycle. In the GemCarbo group, carboplatin was administered either at area under the curve (AUC) 4–5 on day 1 or as a split-dose schedule of AUC 2–2.5 on days 1 and 8, together with gemcitabine 1000 mg/m^2^ on days 1 and 8 every 3 weeks.

Treatment was typically planned for up to eight cycles. However, continuation beyond eight cycles was allowed in selected patients who continued to derive clinical benefit and tolerated treatment. Chemotherapy was discontinued in the event of disease progression, death, unacceptable toxicity, clinical deterioration, or patient preference. Dose modifications, treatment delays, and regimen selection were determined by the primary oncologist based on the patient’s performance status, organ function, baseline laboratory results, and overall clinical condition.

After discontinuation of first-line chemotherapy, subsequent systemic treatment or best supportive care was considered at the discretion of the treating physician. Decisions regarding later-line therapy were individualized according to performance status, residual organ function, prior treatment tolerance, patient preference, and drug availability.

### 2.3. Outcomes and Assessments

The primary objective of this study was to compare OS between patients treated with GemCis and those treated with GemCarbo as first-line therapy for unresectable locally advanced or metastatic BTC. Secondary objectives included comparisons of progression-free survival (PFS), objective response rate (ORR), disease control rate (DCR), and selected safety outcomes between the two treatment groups.

OS was defined as the time from initiation of first-line chemotherapy to death from any cause. Patients who were alive at the last follow-up were censored on the date of their most recent clinical visit. Vital status was verified using medical records and cross-checked with the Thai Social Security Death Index database. PFS was defined as the interval from initiation of first-line chemotherapy to radiologically documented disease progression or death from any cause, whichever occurred first. Patients without documented progression or death were censored on the date of their last disease assessment. Tumor response was evaluated according to the Response Evaluation Criteria in Solid Tumors version 1.1. Computed tomography of the chest and abdomen was generally performed every 2–3 months during treatment, or earlier if clinically indicated, according to routine clinical practice.

The ORR was defined as the proportion of patients who achieved complete or partial response as their best overall response. The DCR was defined as the proportion of patients who achieved complete response, partial response, or stable disease as their best overall response. Safety outcomes were limited to objective laboratory-based adverse events that could be reliably retrieved from retrospective medical records, including acute kidney injury, neutropenia, anemia, and thrombocytopenia. The severity of these events was graded according to the Common Terminology Criteria for Adverse Events version 5.0.

### 2.4. Statistical Analysis

Baseline characteristics were summarized using descriptive statistics. Continuous variables were reported as medians with interquartile ranges (IQRs) or means with standard deviations, as appropriate, whereas categorical variables were presented as frequencies and percentages. Treatment response and safety outcomes were summarized as frequencies and percentages.

Survival outcomes were analyzed using the Kaplan–Meier method, and survival curves were compared between treatment groups using the log-rank test. OS and PFS were reported as medians with corresponding 95% confidence intervals (CIs). Cox proportional hazard regression models were used to estimate hazard ratios (HRs) and 95% CIs, where applicable.

To reduce potential treatment selection bias, propensity score matching was performed. Propensity scores were estimated using baseline variables considered clinically relevant to treatment allocation, including age, sex, ECOG PS, BMI, primary tumor location, advanced disease status at initiation of first-line palliative chemotherapy, and baseline creatinine clearance. Advanced disease status was categorized as unresectable locally advanced disease, de novo metastatic disease, or recurrent metastatic disease. Patients treated with GemCarbo were matched 1:1 to those treated with GemCis using a nearest-neighbor matching algorithm without replacement. No caliper was applied. Covariate balance before and after matching was assessed using standardized mean differences, and balance diagnostics were summarized in a Love plot. Baseline characteristics were reassessed after matching to evaluate balance between the treatment groups.

All statistical analyses were performed using R software version 4.5.3. All statistical tests were two-sided, and a *p*-value of < 0.05 was considered statistically significant.

## 3. Results

### 3.1. Baseline Characteristics

The study included 154 patients with unresectable locally advanced or metastatic BTC, of whom 95 received GemCis and 59 received GemCarbo as first-line palliative chemotherapy. Baseline characteristics are shown in Table 1. The mean age was 60.4 years, 50.6% of patients were male, and most patients had an ECOG PS of 0–1. Intrahepatic cholangiocarcinoma was the most common primary tumor site, and de novo metastatic disease was the most frequent disease status at treatment initiation. Patients in the GemCarbo group were significantly older and had poorer baseline renal function than those in the GemCis group. The mean age was 63.8 years in the GemCarbo group compared with 58.3 years in the GemCis group, and the median creatinine clearance was 48.5 versus 59.5 mL/min, respectively. The proportion of patients with creatinine clearance ≥60 mL/min was also lower in the GemCarbo group. Other baseline characteristics were generally similar between the treatment groups.

Propensity score matching yielded a matched cohort of 118 patients (59 in each treatment group). Baseline characteristics after propensity score matching, together with standardized mean differences before and after matching, are provided in Table S1, and the overall covariate balance is shown in Figure S1. After matching, baseline characteristics, including age, sex, ECOG PS, BMI, primary tumor site, and advanced disease status at the time of treatment initiation, were more comparable between the GemCis and GemCarbo groups. The categorical distribution of CrCl ≥ 60 mL/min was similar between groups, although some residual imbalance remained when CrCl was assessed as a continuous variable.

### 3.2. Treatment Information

Treatment exposure and subsequent therapies are shown in Table 2. Compared with the GemCis group, patients in the GemCarbo group received fewer cycles of first-line chemotherapy and were more likely to undergo initial dose reduction. Among patients treated with GemCarbo, 48 patients (81.4%) received carboplatin AUC 4–5 on day 1, whereas 11 patients (18.6%) received split-dose carboplatin AUC 2–2.5 on days 1 and 8. Disease progression was the leading cause of treatment discontinuation in both groups, followed by performance status deterioration and treatment completion. Patients treated with GemCis were more likely than those treated with GemCarbo to receive subsequent systemic therapy, including both second- and third-line chemotherapies. Details of subsequent systemic therapies are provided in Table S2.

### 3.3. Efficacy Outcomes

#### 3.3.1. Overall Survival

The median observation time for the overall cohort was 8.5 months (IQR, 4.5–14.3 months). During this period, 127 deaths occurred among 154 patients. The median OS for the entire cohort was 9.43 months (95% CI, 8.44–11.3). Estimated OS rates at 12 and 24 months were 39.3% and 16.0%, respectively.

**Figure 1:**
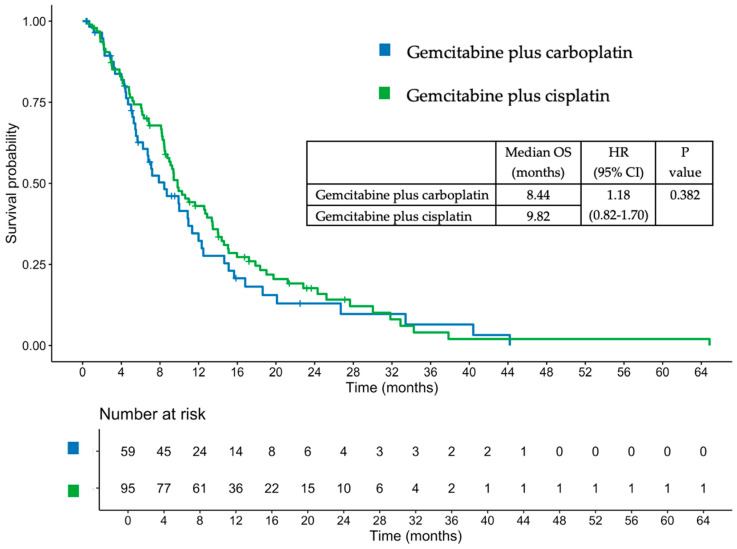
Kaplan–Meier curves for overall survival according to first-line chemotherapy regimen in the overall cohort.

When stratified by treatment regimen, median OS was 8.44 months (95% CI, 6.24–12.0) in the GemCarbo group and 9.82 months (95% CI, 8.54–13.4) in the GemCis group (Figure 1). Although OS was numerically shorter in the GemCarbo group, the difference between groups was not statistically significant (HR, 1.18; 95% CI, 0.82–1.70; *p* = 0.382). In multivariable Cox regression analysis of the overall cohort, GemCarbo was not independently associated with worse OS compared with GemCis after adjustment for baseline clinical factors, including age, ECOG PS, CA19-9 level, albumin level, disease extent, CrCl, and primary tumor site (adjusted HR, 1.07; 95% CI, 0.70–1.62; *p* = 0.760).

#### 3.3.2. Overall Survival After Propensity Score Matching

**Figure 2:**
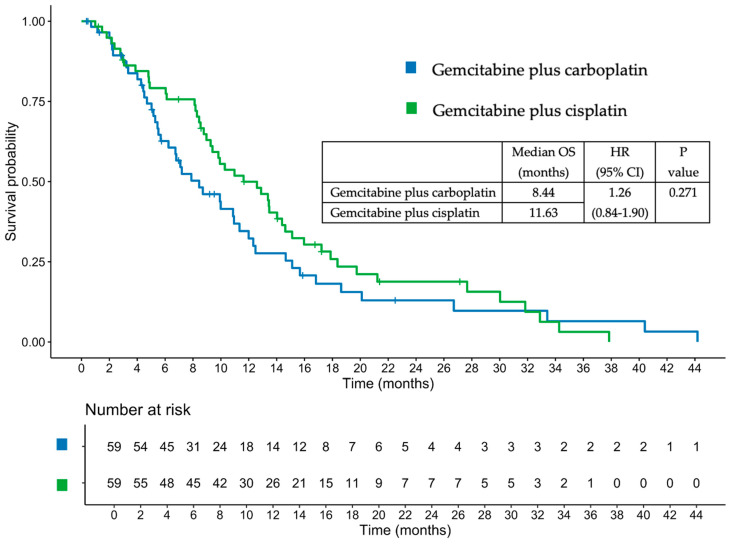
Kaplan–Meier curves for overall survival according to first-line chemotherapy regimen after propensity score matching.

In the propensity score–matched cohort, median OS was 8.44 months (95% CI, 6.24–12.0) in the GemCarbo group and 11.63 months (95% CI, 9.26–14.6) in the GemCis group (Figure 2). Although median OS remained numerically shorter in the GemCarbo group, the difference was not statistically significant (HR, 1.26; 95% CI, 0.84–1.90; *p* = 0.271).

#### 3.3.3. Progression-Free Survival

At the time of analysis, 145 PFS events had occurred among 154 patients. The median PFS for the overall cohort was 4.99 months (95% CI, 3.98–5.98).

**Figure 3:**
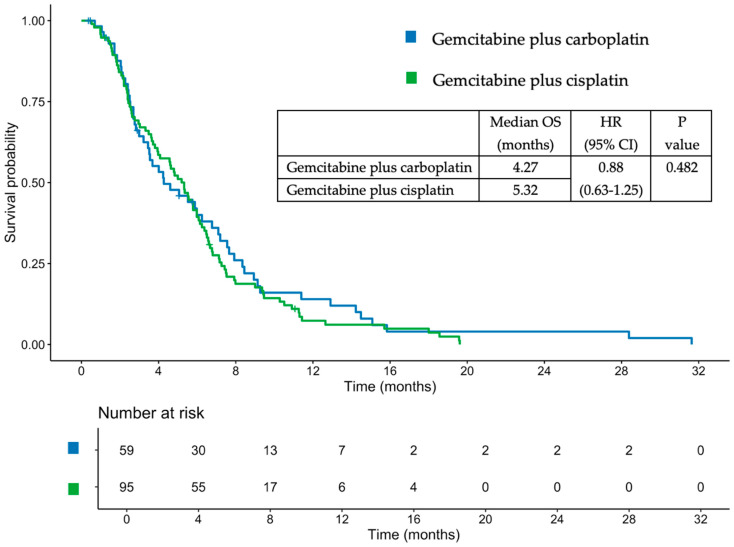
Kaplan–Meier curves for progression-free survival according to first-line chemotherapy regimen in the overall cohort.

When stratified by treatment regimen, median PFS was 4.27 months (95% CI, 3.45–7.10) in the GemCarbo group and 5.32 months (95% CI, 3.98–6.08) in the GemCis group (Figure 3). There was no statistically significant difference in PFS between the two treatment groups (HR, 0.88; 95% CI, 0.63–1.25; *p* = 0.482). In multivariable Cox regression analysis for PFS, GemCarbo was not independently associated with worse PFS compared with GemCis after adjustment for the same baseline covariates (adjusted HR, 0.91; 95% CI, 0.62–1.32; *p* = 0.612).

#### 3.3.4. Progression-Free Survival After Propensity Score Matching

**Figure 4:**
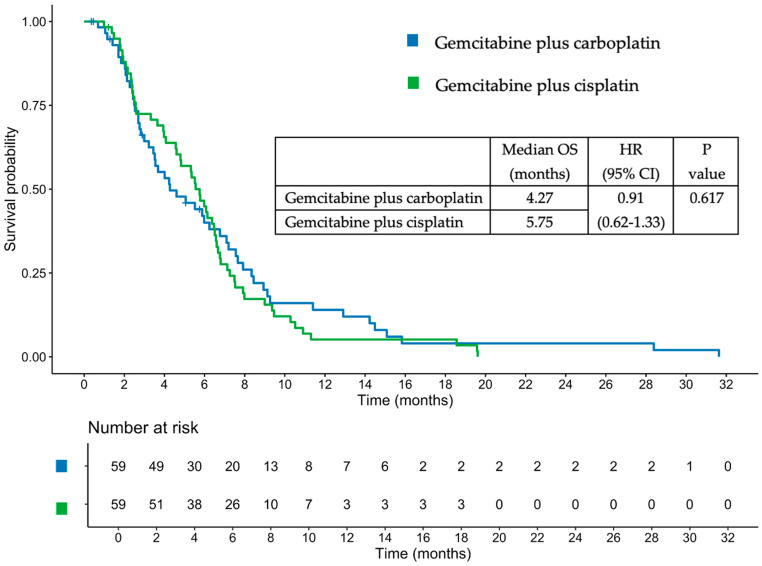
Kaplan–Meier curves for progression-free survival according to first-line chemotherapy regimen after propensity score matching.

In the propensity score–matched cohort, median PFS was 4.27 months (95% CI, 3.45–7.10) in the GemCarbo group and 5.75 months (95% CI, 4.60–6.57) in the GemCis group (Figure 4). The difference between groups was not statistically significant after matching (HR, 0.91; 95% CI, 0.62–1.33; *p* = 0.617).

#### 3.3.5. Tumor Response

Tumor response data are presented in Table 3. Response evaluations were available for 85 patients (89.5%) in the GemCis group and 49 patients (83.1%) in the GemCarbo group. No complete responses were observed. The ORR in the overall treatment population was 10.5% with GemCis and 6.8% with GemCarbo. Among patients with evaluable response data, the ORR was 11.8% and 8.2%, respectively. Stable disease was the most common best response in both groups, resulting in similar disease control rates among evaluable patients (70.6% in the GemCis group and 71.4% in the GemCarbo group).

#### 3.3.6. Selected Safety Outcomes

Selected laboratory-based safety outcomes are listed in Table 4. Increased serum creatinine was more common in the GemCis group than in the GemCarbo group (12.6% vs. 1.7%), including a grade ≥ 3 creatinine increase in 4.2% of patients receiving GemCis and none receiving GemCarbo. Hematologic toxicities were broadly similar between groups. Neutropenia occurred in 27.4% of patients in the GemCis group and 28.8% in the GemCarbo group, whereas anemia occurred in 57.9% and 59.3%, respectively. Thrombocytopenia was numerically more frequent in the GemCarbo group than in the GemCis group (22.0% vs. 15.8%). Grade ≥ 3 hematologic toxicities were uncommon in both treatment groups.

## 4. Discussion

In this real-world retrospective cohort study of patients with unresectable locally advanced or metastatic BTC treated with first-line gemcitabine-platinum chemotherapy, median OS and PFS were numerically shorter in the GemCarbo group, although the differences were not statistically significant in either the overall or propensity score–matched cohorts. These findings suggest that GemCarbo may represent a feasible alternative platinum-based regimen for patients who are less suitable for cisplatin in routine clinical practice. Nevertheless, this study was not designed to establish formal equivalence or non-inferiority, and clinically meaningful differences cannot be excluded.

GemCis has been the standard chemotherapy backbone for advanced BTC since the ABC-02 trial [7], which reported a median OS of 11.7 months and median PFS of 8.0 months with GemCis. More recently, TOPAZ-1 and KEYNOTE-966 established durvalumab plus GemCis and pembrolizumab plus GemCis as first-line standards for eligible patients [9,11], whereas outcomes in the GemCis control arm remained broadly consistent with historical GemCis data [19,20,21]. In the present study, median OS with GemCis was 9.82 months in the overall cohort and 11.63 months in the propensity score–matched cohort. The matched result is comparable to those reported in pivotal clinical trials, whereas the shorter survival in the overall cohort likely reflects the broader real-world population, including patients treated outside trial eligibility criteria [22].

The choice between cisplatin and carboplatin remains relevant in daily clinical practice. Cisplatin may be unsuitable for patients with impaired renal function, frailty, hearing impairment, neuropathy, or a limited ability to tolerate hydration [12,13,23]. This pattern was evident in our study, in which patients who received GemCarbo were older and had lower baseline creatinine clearance than those who received GemCis. They also received fewer treatment cycles and more frequent initial dose reductions. These differences suggest that GemCarbo was preferentially selected for patients who were considered less suitable for cisplatin, which is an important source of confounding in any retrospective comparison. After propensity score matching, the direction of survival remained unchanged, and no significant differences in OS or PFS were observed. Evidences from other tumor types suggests that the relative role of cisplatin and carboplatin is disease- and context-dependent. In advanced non-small-cell lung cancer, carboplatin-based regimens have shown similar OS to cisplatin-based regimens [16]. In ovarian cancer, carboplatin plus paclitaxel demonstrated comparable efficacy with better tolerability than cisplatin plus paclitaxel [17]. Conversely, cisplatin remains difficult to replace in selected settings, such as cisplatin-eligible urothelial carcinoma [18]. These observations support the use of carboplatin as an alternative when cisplatin is unsuitable, but not as a universally interchangeable substitute for cisplatin.

To the best of our knowledge, previous studies evaluating GemCarbo in advanced BTC have generally been single-arm and relatively small. In a phase II study of GemCarbo in advanced BTC, Williams et al. reported a median OS of 10.6 months, median PFS of 7.8 months, and ORR of 31.1% [15]. A retrospective study from India focusing on advanced intrahepatic cholangiocarcinoma reported a median OS of 10.3 months, PFS of 5.3 months, and ORR of 48.36% with GemCarbo [24]. More recently, a single-center UK retrospective study in cisplatin-ineligible patients reported a median OS of 8.97 months and median PFS of 5.88 months [14]. The median OS of 8.44 months observed with GemCarbo in our study is within the range of these prior reports, particularly considering that our GemCarbo group included older patients and those with poorer renal function.

The PFS in our cohort was shorter than that reported in the ABC-02 trial and some previous GemCarbo studies [7,14,15]. Several factors could explain this finding. Our study included patients treated in routine clinical practice rather than in a clinical trial, and the imaging intervals were not strictly protocol-driven. In addition, treatment intensity was lower in the GemCarbo group. Despite these differences, PFS did not significantly differ between GemCarbo and GemCis before or after matching. Tumor response rates were modest in both groups. The ORR among evaluable patients was 11.8% with GemCis and 8.2% with GemCarbo, which was lower than those reported in several prospective studies [14,15,24]. This discrepancy may be related to real-world response assessments, patient selection, treatment exposure, or disease heterogeneity. However, the DCR was similar between the two groups, suggesting that disease stabilization was achieved in a comparable proportion of evaluable patients.

The safety findings were consistent with the expected toxicity profiles of cisplatin and carboplatin [7,14]. Increased serum creatinine was more frequently observed with GemCis. This supports the practical rationale for selecting carboplatin in patients with reduced renal reserve or concern for cisplatin-related nephrotoxicity. Hematologic toxicities, including neutropenia, anemia, and thrombocytopenia, were broadly similar between treatment groups. Prior GemCarbo studies have reported hematologic toxicity as the main adverse event profile, particularly thrombocytopenia, neutropenia, and anemia [14,15,24]. However, our safety analysis was restricted to laboratory-based events. Symptom-based toxicities such as nausea, vomiting, neuropathy, fatigue, and anorexia were not systematically recorded and could not be reliably assessed.

This study should be interpreted in the context of contemporary treatment standards. Chemoimmunotherapy with GemCis plus durvalumab or pembrolizumab is now recommended for eligible patients with advanced BTC [5,6,25,26]. Nevertheless, access to immunotherapy remains variable across healthcare systems, and some patients are unsuitable candidates for cisplatin-based chemoimmunotherapy due to renal impairment, frailty, comorbidities, or other contraindications. Therefore, real-world data on alternative platinum partners remain clinically relevant, particularly in settings where treatment decisions must balance efficacy, tolerability, organ function, and access to newer agents.

This study has limitations. It was retrospective and conducted at a single institution. Treatment selection was not randomized and was influenced by physician judgment, renal function, patient fitness, and expected tolerability. Although propensity score matching reduced measured baseline imbalances, unmeasured confounding remains possible. Because no formal sample size or power calculation was performed, the matched comparison may have been underpowered to exclude a clinically meaningful survival difference, as reflected by the wide 95%CIs. The study period was long and included patients who were treated before the routine incorporation of immune checkpoint inhibitors into first-line therapy. Safety analyses were limited to laboratory-based adverse events, whereas symptom-based toxicities such as nausea, vomiting, neuropathy, hearing impairment, fatigue, and anorexia were not systematically documented and could not be comprehensively assessed. Therefore, the comparative tolerability of GemCarbo and GemCis should be interpreted with caution. Finally, subsequent systemic therapy was more frequently administered in the GemCis group, which may have contributed to the numerically longer OS observed with GemCis. In contrast, PFS is less affected by subsequent therapy and showed no statistically significant difference between treatment groups, supporting a cautious interpretation of the OS findings.

Despite those limitations, this study provides clinically relevant real-world comparative data on GemCarbo versus GemCis in advanced BTC. Unlike most previous studies, which were single-arm cohorts, this study directly compared the two regimens and used propensity score matching to reduce measured treatment selection bias. Our findings suggest that GemCarbo may be considered an alternative gemcitabine-platinum regimen. Prospective studies are required to determine whether GemCarbo provides formally non-inferior outcomes compared with GemCis.

## 5. Conclusions

GemCarbo demonstrated no statistically significant difference in OS or PFS compared with GemCis in patients with unresectable locally advanced or metastatic BTC, even after propensity score matching. GemCarbo was more commonly used in older patients and those with reduced renal function, reflecting its role as a practical alternative to cisplatin in real-world clinical practice. These findings suggest that GemCarbo may be considered a feasible treatment option when cisplatin is unsuitable. However, given the retrospective design, limited sample size, and wide 95% CIs, these findings should not be interpreted as evidence of equivalence or non-inferiority. Further prospective validation is warranted.

## Figures and Tables

**Table 1:** Baseline characteristics.

Characteristics	GemCis (*n* = 95)	GemCarbo (*n* = 59)	Total (*n* = 154)
Mean age, years (SD) *	58.3 (9.4)	63.8 (10.8)	60.4 (10.3)
Age ≥ 65 years, *n* (%) *	20 (21.1)	25 (42.4)	45 (29.2)
Sex, *n* (%)			
Male	53 (55.8)	25 (42.4)	78 (50.6)
Female	42 (44.2)	34 (57.6)	76 (49.4)
ECOG PS, *n* (%)			
0–1	87 (91.6)	55 (93.2)	142 (92.2)
≥2	8 (8.4)	4 (6.8)	12 (7.8)
BMI, *n* (%)			
<18.5 kg/m^2^	16 (16.8)	19 (32.2)	35 (22.7)
18.5–22.9 kg/m^2^	42 (44.2)	25 (42.4)	67 (43.5)
≥23 kg/m^2^	37 (38.9)	15 (25.4)	52 (33.8)
Comorbidity, *n* (%)			
Cirrhosis	4 (4.2)	6 (10.2)	10 (6.5)
HBV infection	6 (6.3)	4 (6.8)	10 (6.5)
HCV infection	3 (3.2)	0 (0)	3 (1.9)
Laboratory values			
TB, mg/dL	0.7 (0.4, 1.1)	0.6 (0.5, 1.1)	0.7 (0.4, 1.1)
AST, U/L	37 (23.5, 57.0)	37 (24.5, 58.0)	37 (24.0, 57.0)
ALT, U/L	24 (17.5, 45.5)	20 (16.0, 45.5)	23 (17.0, 45.8)
ALP, U/L	175 (115.5, 323.0)	159 (109.5, 291.0)	160 (112.5, 318.8)
Albumin, g/dL (IQR)	3.9 (3.5, 4.2)	3.9 (3.5, 4.3)	3.9 (3.4, 4.3)
Albumin ≥ 3.5 g/dL, *n* (%)	73 (76.8)	38 (64.4)	111 (72.1)
CA 19-9, U/mL (IQR)	100 (15.9, 1186.5)	60 (3.5, 3470.8)	85.1 (10.0, 1393.8)
CA 19-9 ≥ 100 U/mL, *n* (%)	47 (49.5)	27 (45.8)	74 (48.1)
CrCl, mL/min (IQR) *	59.5 (49.4, 71.1)	48.5 (37.6, 58.4)	55.1 (44.7, 69.5)
CrCl ≥ 60 mL/min, *n* (%) *	45 (47.4)	13 (22.0)	58 (37.7)
Primary tumor site, *n* (%)			
Intrahepatic	46 (48.4)	32 (54.2)	78 (50.6)
Extrahepatic	29 (30.5)	17 (28.8)	46 (29.9)
Gallbladder	15 (15.8)	7 (11.9)	22 (14.3)
Ampulla of Vater	5 (5.3)	3 (5.1)	8 (5.2)
Advanced disease status, *n* (%)			
Recurrent metastasis	19 (20.0)	15 (25.4)	34 (22.1)
De novo metastasis	70 (73.7)	39 (66.1)	109 (70.8)
Unresectable locally advanced	6 (6.3)	5 (8.5)	11 (7.1)
Number of organ metastasis, *n* (%)			
1	52 (54.7)	27 (45.8)	79 (51.3)
2	20 (21.1)	20 (33.9)	40 (26.0)
≥3	17 (17.9)	7 (11.9)	24 (15.6)
Organ metastasis, *n* (%)			
Liver	47 (49.5)	31 (52.5)	78 (50.6)
Lung	25 (26.3)	13 (22.0)	38 (24.7)
Distant lymph node	41 (43.2)	31 (52.5)	72 (46.8)
Peritoneum	23 (24.2)	10 (16.9)	33 (21.4)
Bone	9 (9.5)	5 (8.5)	14 (9.1)
Adrenal	5 (5.3)	0 (0)	5 (3.2)
Pleura	1 (1.1)	0 (0)	1 (0.6)
Previous treatment, *n* (%)			
Surgery	20 (21.1)	19 (32.2)	39 (25.3)
Adjuvant chemotherapy	8 (8.4)	6 (10.2)	14 (9.1)
Biliary drainage	40 (42.1)	22 (37.3)	62 (40.3)

GemCis, gemcitabine plus cisplatin; GemCarbo, gemcitabine plus carboplatin; ECOG PS, Eastern Cooperative Oncology Group performance status; BMI, body mass index; HBV, hepatitis B virus; HCV, hepatitis C virus; TB, total bilirubin; AST, aspartate aminotransferase; ALT, alanine aminotransferase; ALP, alkaline phosphatase; CrCl, creatinine clearance. * *p* < 0.05 indicates a statistically significant difference between the GemCis and GemCarbo groups.

**Table 2:** Treatment information.

Treatment Information	GemCis (*n* = 95)	GemCarbo (*n* = 59)
Number of cycles (IQR) *	6 (3, 8)	4 (2, 6)
Initial dose reduction *	26 (27.4)	28 (47.5)
Discontinuation, *n* (%)		
Progressive disease	59 (62.1)	23 (39.0)
Complete treatment	13 (13.7)	13 (22.0)
PS deterioration	16 (16.8)	13 (22.1)
Infection	2 (2.1)	4 (6.8)
Death	3 (3.2)	1 (1.7)
Loss of follow-up	2 (2.1)	5 (8.5)
Subsequent treatment, *n* (%)		
Second-line chemotherapy *	53 (55.8)	18 (30.5)
Third-line chemotherapy *	20 (21.1)	4 (6.8)

GemCis, gemcitabine plus cisplatin; GemCarbo, gemcitabine plus carboplatin; PS, performance status. * *p* < 0.05 indicates a statistically significant difference between the GemCis and GemCarbo groups.

**Table 3:** Tumor response.

	GemCis (*n* = 95)	GemCarbo (*n* = 59)
Evaluable patients, *n* (%)	85 (89.5)	49 (83.1)
Complete response, *n* (%)	0 (0)	0 (0)
Partial response, *n* (%)	10 (10.5)	4 (6.8)
Stable disease, *n* (%)	50 (52.6)	31 (52.5)
Progressive disease, *n* (%)	25 (26.3)	14 (23.7)
ORR in the overall population, *n* (%)	10 (10.5)	4 (6.8)
ORR in the evaluable patients, *n* (%)	10 (11.8)	4 (8.2)
DCR in the overall population, *n* (%)	60 (63.2)	35 (59.3)
DCR in the evaluable patients, *n* (%)	60 (70.6)	35 (71.4)

GemCis, gemcitabine plus cisplatin; GemCarbo, gemcitabine plus carboplatin; ORR, objective response rate; DCR, disease control rate.

**Table 4:** Selected safety outcomes.

Adverse Event	GemCis (*n* = 95)			GemCarbo (*n* = 59)		
	All Grades	Grades 1–2	Grades 3–4	All Grades	Grades 1–2	Grades 3–4
Creatinine increased	12 (12.6)	8 (8.4)	4 (4.2)	1 (1.7)	1 (1.7)	0
Neutropenia	26 (27.4)	24 (25.3)	2 (2.1)	17 (28.8)	16 (27.1)	1 (1.7)
Anemia	55 (57.9)	53 (55.8)	2 (2.1)	35 (59.3)	35 (59.3)	0
Thrombocytopenia	15 (15.8)	14 (14.7)	1 (1.1)	13 (22.0)	11 (18.6)	2 (3.4)

## Data Availability

The raw data supporting the conclusions of this article will be made available by the authors on request.

## Supplementary Materials

The following supporting information can be downloaded at: https://www.mdpi.com/article/10.3390/life16071150/s1, Table S1: Baseline characteristics before and after propensity score matching with standardized mean difference; Table S2: Subsequent systemic therapies after discontinuation of first-line chemotherapy; Figure S1: Love plot showing absolute standardized mean differences for baseline covariates before and after propensity score matching.

## Abbreviations

The following abbreviations are used in this manuscript:

BTC	Biliary tract cancer
GemCis	Gemcitabine plus cisplatin
GemCarbo	Gemcitabine plus carboplatin
AUC	Area under the curve
OS	Overall survival
PFS	Progression-free survival
ORR	Objective response rate
DCR	Disease control rate
IQR	Interquartile range
CI	Confidence interval
HR	Hazard ratio
BMI	Body mass index
ECOG PS	Eastern Cooperative Oncology Group performance status
SD	Standard deviation
HBV	Hepatitis B virus
HCV	Hepatitis C virus
TB	Total bilirubin
AST	Aspartate aminotransferase
ALT	Alanine aminotransferase
ALP	Alkaline phosphatase
CrCl	Creatinine clearance
