# Supplementary material for: Choosing the Platinum Partner in Advanced Biliary Tract Cancer: A Propensity Score–Matched Real-World Comparison of Gemcitabine Plus Carboplatin Versus Gemcitabine Plus Cisplatin
Source: Life (Basel). 2026 Jul 11;16(7):1150. doi: 10.3390/life16071150 (PMC13413175; doi:10.3390/life16071150)
Supplement: Supplementary file 1 [file life-16-01150-s001.zip › life-4392585-Table S1.pdf]

Table S1 Baseline characteristics after propensity score matching

Characteristics	GemCis (n=59)	GemCarbo (n=59)
Age ≥ 65 years, n (%)	17 (28.8)	25 (42.4)
Sex, n (%)		
Male	32 (54.2)	25 (42.4)
Female	27 (45.8)	34 (57.6)
ECOG PS, n (%)		
0-1	56 (94.9)	55 (93.2)
≥ 2	3 (5.1)	4 (6.8)
BMI, n (%)		
< 18.5 kg/m^2	15 (25.4)	19 (32.2)
18.5–22.9 kg/m^2	26 (44.1)	25 (42.4)
≥ 23 kg/m^2	18 (30.5)	15 (25.4)
CrCl ≥ 60 mL/min, n (%)	14 (23.7)	13 (22.0)
Primary tumor site, n (%)		
Intrahepatic	30 (50.8)	32 (54.2)
Extrahepatic	16 (27.1)	17 (28.8)
Gallbladder	10 (16.9)	7 (11.9)
Ampulla of Vater	3 (5.1)	3 (5.1)
Advanced disease status, n (%)		
Recurrent metastasis	15 (25.4)	15 (25.4)
De novo metastasis	39 (66.1)	39 (66.1)
Unresectable locally advanced	5 (8.5)	5 (8.5)

GemCis, gemcitabine plus cisplatin; GemCarbo, gemcitabine plus carboplatin; ECOG PS, Eastern Cooperative Oncology Group performance status; BMI, body mass index; CrCl, creatinine clearance.
