# Supplementary material for: Choosing the Platinum Partner in Advanced Biliary Tract Cancer: A Propensity Score–Matched Real-World Comparison of Gemcitabine Plus Carboplatin Versus Gemcitabine Plus Cisplatin
Source: Life (Basel). 2026 Jul 11;16(7):1150. doi: 10.3390/life16071150 (PMC13413175; doi:10.3390/life16071150)
Supplement: Supplementary file 1 [file life-16-01150-s001.zip › life-4392585-Table S2 & Figure S1.pdf]

Table S2 Subsequent systemic therapies after discontinuation of first-line chemotherapy

Subsequent therapy	GemCis (n = 95)	GemCarbo (n = 59)
Second-line therapy, n (%)	53 (55.8)	18 (30.5)
FOLFOX	28 (29.5)	7 (11.9)
CAPOX	6 (6.3)	1 (1.7)
Carboplatin plus 5-FU	13 (13.7)	5 (8.5)
Carboplatin plus gemcitabine	1 (1.1)	1 (1.7)
Cisplatin plus 5-FU	1 (1.1)	1 (1.7)
Cisplatin plus gemcitabine	1 (1.1)	0 (0)
5-FU	2 (2.1)	2 (3.4)
TS-one	0 (0)	1 (1.7)
Clinical trial	1 (1.1)	0 (0)
Third-line therapy, n (%)	20 (21.1)	4 (6.8)
FOLFIRI	14 (14.7)	1 (1.7)
FOLFOX	2 (2.1)	1 (1.7)
CAPOX	1 (1.1)	0 (0)
Irinotecan	0 (0)	1 (1.7)
Paclitaxel	0 (0)	1 (1.7)
Trastuzumab deruxtecan	1 (1.1)	0 (0)
Carboplatin plus 5-FU	1 (1.1)	0 (0)
Carboplatin plus gemcitabine	1 (1.1)	0 (0)

GemCis, gemcitabine plus cisplatin; GemCarbo, gemcitabine plus carboplatin; FOLFOX, folinic acid plus 5-fluorouracil and oxaliplatin; CAPOX, capecitabine plus oxaliplatin; 5-FU, 5-fluorouracil; TS-one, tegafur plus gimeracil and oteracil; FOLFIRI, folinic acid plus 5-fluorouracil and irinotecan.

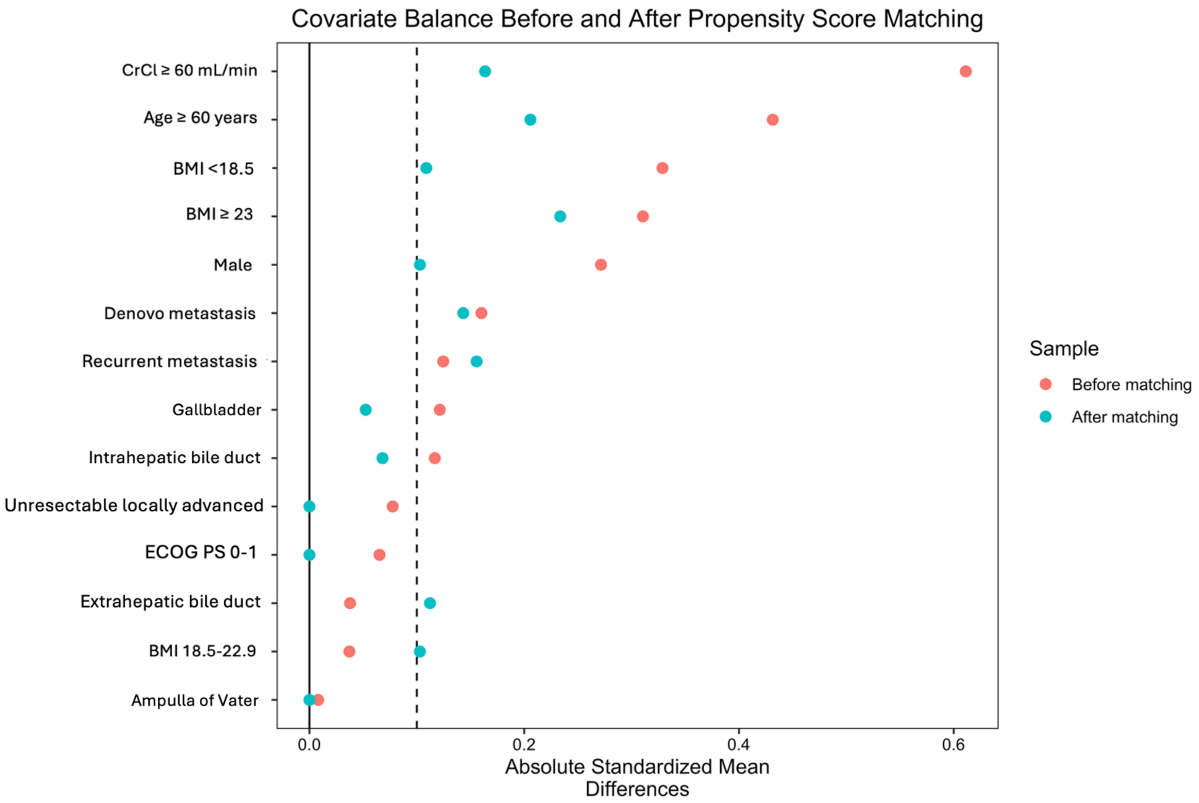

Figure S1 Love plot showing absolute standardized mean differences for baseline covariates before and after propensity score matching. The dashed vertical line indicates a standardized mean difference of 0.1.
